# Clinical and radiological course of asymptomatic and hemodynamically stable moyamoya disease: a systematic review and meta-analysis

**DOI:** 10.3389/fneur.2025.1626817

**Published:** 2025-07-08

**Authors:** Yang Chen, Zixuan Zhou, Bingyang Qin, Yan Liang, Peize Li, Ziao Li, Yu Chen, Ren Li, Biao Yang, Xiaogang Wang, Yongqiang Wu, Xiaolong Guo, Huidong Zhang, Geng Guo

**Affiliations:** ^1^Department of Neurosurgery, First Hospital of Shanxi Medical University, Taiyuan, China; ^2^Cerebrovascular Disease Center, First Hospital of Shanxi Medical University, Taiyuan, China; ^3^Center for Cerebrovascular Diseases Research, Shanxi Medical University, Taiyuan, China; ^4^MOE Key Laboratory of Coal Environmental Pathogenicity and Prevention, Shanxi Medical University, Taiyuan, China; ^5^Department of Emergency, First Hospital of Shanxi Medical University, Taiyuan, China; ^6^School of Basic Medicine, Shanxi Medical University, Taiyuan, China; ^7^Department of Neurosurgery, Beijing Tiantan Hospital, Capital Medical University, Beijing, China; ^8^National Clinical Research Center for Neurological Diseases, Beijing, China; ^9^Department of Environmental Health, School of Public Health, Shanxi Medical University, Taiyuan, China; ^10^Key Laboratory of Environment and Female Reproductive Health, The Eighth Affiliated Hospital, Sun Yat-sen University, Guangzhou, Shenzhen, China

**Keywords:** moyamoya disease, asymptomatic, hemodynamically stable, clinical course, radiological course

## Abstract

**Background:**

Moyamoya disease (MMD) is an idiopathic, chronic intracranial vascular stenosis and occlusion disease. However, there is currently a lack of comprehensive analysis on the clinical and radiological course of asymptomatic MMD (AMMD) and hemodynamically stable MMD (HSMMD).

**Data source:**

We conducted a comprehensive literature search using major bibliographic indexing databases, including Embase, Medline, PubMed, Web of Science, and Cochrane Library.

**Methods:**

This systematic review was conducted based on the PRISMA guidelines. The quality of the included studies was accessed using the Methodological Index for Non-Randomized Studies (MINORS). Effect sizes were pooled with a random-effects model. Heterogeneity between studies was estimated via the I^2^ test. Publication bias was assessed with Egger’s test. The registration code is CRD42023444432.

**Result:**

A total of seven AMMD studies were included in a meta-analysis, involving 393 patients and 649 hemispheres. Three HSMMD studies were all from the same institution. The pooled rate for clinical progression, hemorrhagic stroke, ischemic stroke, transient ischemic attack (TIA), and radiological progress of conservative group was 10% (95% CI 4.9–15.1%), 3.8% (95% CI 0.4–7.2%), 0.7% (95% CI 0–2.3%), 3.6% (95% CI 0.6–6.6%), and 15.6% (95% CI 10.2–22.1%), respectively. The pooled rate for stroke, TIA, and radiological progress of the surgical group was 3.7% (95% CI 0–10.8%), 0.2% (95% CI 0–3.0%), and 4.8% (95% CI 0–10.5%), respectively. Revascularization did not show a protective effect on TIA and radiological progression for AMMD.

**Conclusion:**

AMMD and HSMMD present a concerning risk of clinical and radiological progression over a follow-up period of more than 2 years. Further high-quality studies are needed to optimize treatment strategies.

**Systematic review registration:**

https://www.crd.york.ac.uk/prospero/display_record.php?RecordID=444432, CRD42023444432.

## 1 Introduction

Moyamoya disease (MMD) is an idiopathic chronic intracranial vascular steno-occlusive disease characterized by progressive stenosis of the terminal segments of the internal carotid arteries bilaterally and/or partial stenosis or occlusion of the proximal portions of the anterior cerebral arteries and the middle cerebral arteries and formation of an abnormal network of vessels at the base of the skull (1, 2). MMD was first reported in Japan in the 1950s and has gradually gained worldwide recognition. The annual morbidity of MMD is 0.5–1.5 per 100,000 in East Asia and 0.1 in other regions (3). Based on hemodynamic stability, MMD can be classified into hemodynamically unstable MMD and hemodynamically stable MMD (HSMMD). Multiple techniques can be used to assess hemodynamic status, including single-photon emission computed tomography (SPECT), perfusion computed tomography (CT), and dynamic susceptibility contrast magnetic resonance imaging (MRI) (4). However, the majority of the studies use normal or slight decrease in basal perfusion and a decrease in the reserve capacity after acetazolamide challenge at less than 50% of the basal perfusion on SPECT to identify HSMMD (5). According to the occurrence of clinical symptoms, MMD can be classified as asymptomatic MMD (AMMD) and symptomatic MMD. Common clinical manifestations include intracranial hemorrhage, cerebral infarction, transient ischemic attack (TIA), and epileptic seizures, which may occur in some patients. Asymptomatic MMD is a special and important one, which refers to patients who have not experienced hemorrhage, ischemia, and other symptoms of neurological deficits before incidental diagnosis (6). At present, the gold standard for the diagnosis of MMD is still digital subtraction angiography (DSA) (4). Considering the implementation conditions and invasiveness of DSA, perfusion and angiography methods based on CT and MRI are the alternative options. With the continuous popularization of diagnostic technology, the detection rate of AMMD continues to increase (7). In a nationwide survey conducted in Japan in 1994, AMMD accounted for 1.5% of all cases of MMD; however, in a detailed survey conducted in Japan in 2008, this proportion rose to 17.8% (8, 9). Therefore, the actual proportion of AMMD may be higher.

Current treatment options for MMD are categorized as non-surgical (conservative observation and antiplatelet agents) and surgical (surgical revascularization with direct or indirect bypasses or a combination of both). The application of endovascular intervention in MMD has not shown satisfactory results, but it serves as the preferred therapy for posterior circulation aneurysms combined with MMD (10). Revascularization has been shown to be effective in improving the prognosis and reducing the risk of adverse events in patients with symptomatic MMD (11–13). In HSMMD and AMMD patients, the natural history of the disease is uncertain, with a variety of factors influencing it (5); the long-term outcome is unclear, and the short- and long-term benefits of surgical treatment have varied from study to study (13). At present, some theories believe that symptomatic MMD should be treated with surgery regardless of the hemodynamic status. However, due to the inconsistency of hemodynamic status assessment methods and standards, the determination and corresponding treatment of patients with HSMMD still need to be explored (4). Treatments of AMMD still require the support of evidence-based medicine. In the natural course of AMMD, it is crucial to balance the risks of stroke or hemorrhage and the risks of complications caused by treatment for the benefit of patients.

However, there is currently a lack of comprehensive analysis of the natural course and post-treatment process of AMMD and HSMMD. This systematic review and meta-analysis will summarize the existing literature, clarify the natural disease course and post-treatment process of AMMD and HSMMD, and help medical workers and health policymakers formulate optimal treatment options.

## 2 Materials and methods

### 2.1 Search strategy

The present systematic review is written in accordance with the PRISMA statement (14). The review protocol has been registered in the PROSPERO system (CRD42023444432^1^). The eligible articles in Embase, Medline, PubMed, Web of Science, and Cochrane Library databases from the beginning to November 2023 were systematically searched. We conducted the second search in November 2024 to include new published works of literature from 2023 to 2024. Keywords used to construct search terms include “asymptomatic,” “hemodynamically stable,” and “moyamoya disease.” The complete search queries can be obtained in the Supplementary material.

### 2.2 Outcome definitions

The outcomes included in this study were clinical progress, radiological progression, TIA, and stroke incidence during follow-up. Clinical progress is defined as neurological symptoms associated with MMD, including TIA, ischemia, and hemorrhagic symptoms. Radiological progress includes progression in the MMD stage or new anomalies on MRI, CT, or perfusion imaging. Given that the mean follow-up time for most included studies was within 2–5 years, the mortality rate of AMMD was extremely low between this time horizon, and almost all studies did not describe mortality, this systematic review did not include mortality as the primary outcome.

### 2.3 Inclusion and exclusion criteria

The studies included must reach the following criteria: (1) studies reporting the natural or post-treatment clinical course of AMMD or HSMMD; (2) the study should report any of the aforementioned outcomes; (3) either prospective or retrospective study is qualified; (4) if a study reports symptomatic MMD or contains pediatric patients, the data should be represented severally; and (5) the mean or median follow-up time should be more than 12 months.

The exclusion criteria: (1) studies are reviews, letters, meta-analyses, case reports, or comments; (2) the study cohort is all pediatric or adult outcome data in the cohort are inseparable; (3) fewer than 10 patients in the entire cohort; (4) the study contains overlapping cohorts; and (5) non-English published studies. Cohorts from the same institution and overlap period is considered as overlapping cohorts, and only one of them will be included in the meta-analysis.

### 2.4 Data extraction

Four authors independently conducted the retrieval, screening, and data extraction. The disagreements are resolved by consensus or decided by senior researchers. The extracted data included study basic information (year, journal, author, country of publication, and study type), cohort baseline information (number of patients/hemispheres included, age, gender, concomitant disease, Suzuki stage, and follow-up time), and study outcomes. For the overlapping cohort, we will include the higher-quality study. If any data is not available, we will contact the corresponding author to obtain the complete data for analysis.

### 2.5 Quality assessment

Four authors independently conducted quality assessment, and the disagreements were resolved by consensus. Studies were appraised by the methodological index for the non-randomized study (MINORS) scale (15). The scale includes eight assessing items for non-comparative studies and four additional items for comparative studies.

### 2.6 Statistical analysis

Using R and R package “meta” for analysis. Calculate the pooled effect sizes and 95% confidence intervals (CIs) for outcomes. All analyses were performed using a random effect model. Heterogeneity was detected with the I^2^ and 95% CI. Using Egger’s test to evaluate publication bias for pooling ≥5 studies. Data expressed as mean ± standard deviation, mean (range), or number of events (percentage). Other data formats will be marked.

## 3 Results

### 3.1 Screening process

**Figure 1:**
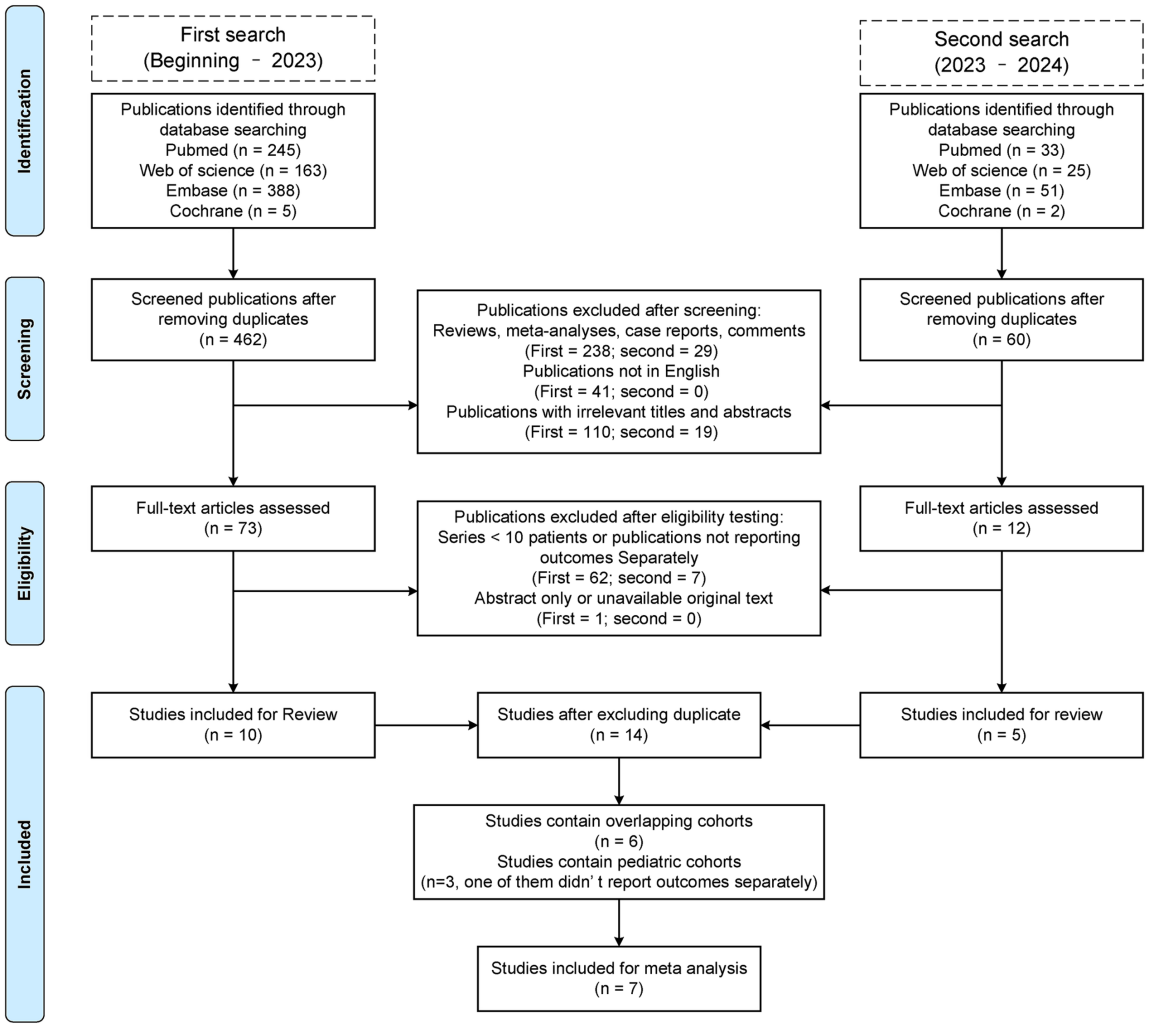
Preferred reporting items for systematic reviews and meta-analysis (PRISMA) flow diagram of this review.

We evaluated a total of 522 unique publications, of which 508 were excluded (Figure 1). We performed data extraction on 14 studies, including six overlapping cohort studies, of which three are HSMMD cohorts (Tables 1, 2) (16). A total of three studies included pediatric patients, with data from one of which is inseparable.

**Table 1:** Baseline information for studies included in meta-analysis.

Study	Year	Journal	Country	Type	No. of patients/hemispheres	Follow-up	S/C
Satoshi et al.	2007	Stroke	JPN	Historical prospective	39/75	43.7 (1–150) m	6/33
Jeyul et al.	2014	Journal of Cerebrovascular and Endovascular Neurosurgery	KOR	Retrospective	42/75	37.3 (7.4–108.7) m	0/42
Kyung et al.	2014	Cerebrovascular Diseases	KOR	Retrospective	40/74	Clinical: median 24 (12–108) m Radiological: median 32 (6–203) m	6/34
Hanhai et al.	2020	World Neurosurgery	CHN	Retrospective	23/48	Surgical: 30.45 ± 4.99 (SE) m Conservative: 39 ± 4.34 (SE) m	11/12
Pui et al.	2022	World Neurosurgery	USA	Retrospective	97/106	Surgical: 4.9 ± 4.6 y Conservative: 6.4 ± 5.9 y	47/50
Yuting et al.	2023	Stroke	CHN	Retrospective	49/89	49.86 (4.41–231.29) m	27/22
Satoshi et al.	2023	Stroke	JPN	Prospective	103/182	Minimum 5 y	0/103

Study	Age (T/S/C)	Gender (M/F)	Diabetes mellitus	Hypertension	Dyslipidemia	Thyroid disease	Intracranial aneurysm	Smoking	Family history of MMD
Satoshi et al.	NR	13/26	NR	NR	NR	NR	NR	NR	NR
Jeyul et al.	41.2 (23–64)	10/32	2 (4.76%)	11 (26.19%)	NR	1 (2.38%)	NR	NR	8 (19.05%)
Kyung et al.	Median 48 (29–76)	14/26	4 (10%)	13 (32.5%)	7 (17.5%)	NR	3 (7.5%)	4 (10%)	4 (10%)
Hanhai et al.	Median 46.4 (30–58)	9/14	1 (4.35%)	8 (34.78%)	2 (8.7%)	1 (4.35%)	4 (17.39%)	7 (30.43%)	0
	49.36 ± 1.75 (SE)	4/7	0	4 (36.36%)	2 (18.18%)	0	3 (27.27%)	5 (45.45%)	0
	43.67 ± 2.31 (SE)	5/7	1 (8.33%)	4 (33.33%)	0	1 (8.33%)	1 (8.33%)	2 (16.67%)	0
Pui et al.	43 ± 13	22/84	26 (24.53%)	52 (49.06%)	34 (32.08%)	NR	13 (12.26%)	45 (42.45%)	NR
	45 ± 14	8/39	15 (31.91%)	23 (48.94%)	16 (34.04%)		8 (17.02%)	22 (46.81%)	
	40 ± 11	14/45	11 (18.64%)	29 (49.15%)	18 (30.51%)		5 (8.47%)	23 (38.98%)	
Yuting et al.	42.35 ± 9.95	25/24	2 (4.08%)	13 (26.53%)	6 (12.24%)	NR	6 (12.24%)	5 (10.2%)	NR
	41.04 ± 8.30	17/10	2 (7.41%)	10 (37.04%)	2 (7.41%)		4 (14.81%)	3 (11.11%)	
	43.95 ± 11.45	8/14	0	3 (13.64%)	4 (18.18%)		2 (9.09%)	2 (9.09%)	
Satoshi et al.	43.8 ± 9.8	32/71	3 (2.91%)	23 (22.33%)	20 (19.42%)	NR	NR	6 (5.83%)	21 (20.39%)

Study	Suzuki stage						PCA	MINORS
	1	2	3	4	5	6		
Satoshi et al.	3 (4.29%)	10 (14.29%)	32 (45.71%)	21 (30%)	2 (2.86%)	2 (2.86%)	NR	17/24
Jeyul et al.	NR						NR	12/16
Kyung et al.	6 (9.68%)	13 (20.97%)	16 (25.81%)	20 (32.26%)	6 (9.68%)	1 (1.61%)	6 (9.68%)	18/24
Hanhai et al.	0*	3* (13.04%)	10* (43.48%)	6* (26.08%)	4* (17.39%)	0*	2	19/24
Pui et al.	2.8 ± 1.1						NR	19/24
	2.8 ± 1.1							
	2.8 ± 1.2							
Yuting et al.	27 (30.34%)		62 (69.66%)				6	18/24
Satoshi et al.	56 (30.77%)			87 (47.80%)			14/182	14/16
	Questionable hemispheres: 39 (21.43)							

S/C, Surgical/Conservative; T/S/C, Total/Surgical/Conservative; M/F, Male/Female; PCA, Posterior cerebral artery; SE, Standard error; IQR, interquartile range; NR, Not reported; m, mouth; y, year; The data are presented as mean ± standard deviation, mean (range), and number (percentage).

* Present worsen grade of the hemisphere.

**Table 2:** Baseline information for overlapping cohorts, HSMMD cohorts, and cohorts containing pediatric patients.

Study	Year	Journal	Country	Type	No. of patients/hemispheres	Follow up	S/C
HSMMD in overlapping cohorts							
Won et al.	2015	JNS	KOR	Retrospective	241/449	82.5 ± 62.9 m	0/241
Chang et al.	2021	Scientific Reports	KOR	Retrospective	243/439	62.0 (6–218) m	0/243
Young et al.	2024	Scientific Reports	KOR	Retrospective	288/288	62.9 ± 46.5 m	0/288
Overlapping cohorts							
Yudai et al.	2020	Stroke	JPN	Retrospective	84/NR	mean 42.2 m	0/84
Ryosuke et al.	2024	World Neurosurgery	JPN	Retrospective	24/NR	Minimum 12 m	0/24
Yong et al.	2024	JAMA Network Open	KOR	Retrospective	15,989/N	median 5.76 [3.00, 9.43] y (IQR)	3,367/12,622
Cohorts containing pediatric patients							
Satoshi et al.	2007	Stroke	JPN	Historical prospective	40/77	43.7 (1–150) m	6/34
Rutao et al.	2017	World Neurosurgery	CHN	Retrospective	61/116	56.32 (11.3–112.62) m	52/9
Yuting et al.	2023	Stroke	CHN	Retrospective	64/119	49.86 (4.41–231.29) m	40/24

Study	Age (T/S/C)	Gender (M/F)	Diabetes mellitus	Hypertension	Dyslipidemia	Thyroid disease	Smoking	Family history of MMD
HSMMD in overlapping cohorts								
Won et al.	41.3 ± 12.0 (18–69)	75/116	NR	73 (30.29%)	30 (12.45%)	12 (4.98%)	NR	19 (7.88%)
Chang et al.	43.7 ± 11.4 (20–74)	61/182	NR	85 (34.98%)	92 (37.86%)	NR	14 (5.76%)	47 (19.34%)
Young et al.	40.8 ± 12.2 (18–71.3)	97/191	33 (11.46%)	92 (31.94%)	53 (18.4%)	9 (3.13%)	NR	55 (19.1%)
Overlapping cohorts								
Yudai et al.	47.8 ± 11.5	30/54	4 (4.76%)	31 (36.9%)	9 (10.71%)	NR	8 (9.52%)	17 (20.24%)
Ryosuke et al.	NR	NR	NR	NR	NR	NR	NR	NR
Yong et al.	44.73 ± 15.24	5,648/10,341	2,190 (13.7%)	5,172 (32.35%)	4,641 (29.03%)	NR	NR	NR
	38.61 ± 13.34	1,108/2,259	389 (11.55%)	867 (25.75%)	776 (23.05%)			
	46.36 ± 15.3	4,540/8,082	1,801 (14.27%)	4,305 (34.11%)	3,865 (30.62%)			
Cohorts containing pediatric patients								
Satoshi et al.	41.4 ± 12.6 (13–67)	13/27	NR	NR	NR	NR	NR	NR
Rutao et al.	26.33 (6–59)	21/40	1 (1.64%)	2 (3.28%)	NR	0	NR	1 (1.64%)
	25.02 (6–52)	20/32	0	1 (1.92%)		0		1 (1.92%)
	33.89 (8–59)	1/8	1 (11.11%)	1 (11.11%)		0		0
Yuting et al.	34.77 ± 16.34	34/30	2 (3.13%)	13 (20.31%)	6 (9.38%)	NR	6 (9.38%)	NR
	30.83 ± 16.37	24/16	2 (5%)	10 (25%)	2 (5%)		4 (10%)	
	41.33 ± 14.00	10/14	0 (0%)	3 (12.5%)	4 (16.67%)		2 (8.33%)	

Study	Suzuki stage						PCA	MINORS
	1	2	3	4	5	6		
HSMMD in overlapping cohorts								
Won et al.	R 3.9 ± 0.5 (*n* = 221, range 2–5), L 3.9 ± 0.6 (*n* = 228, range 2–5)						NR	12/16
Chang et al.	NR						NR	18/24
Young et al.	3.3 ± 1.1 (2–6)						NR	13/16
Overlapping cohorts								
Yudai et al.	69 (82.14%)			15 (17.86%)			13 (15.48%)	18/24
Ryosuke et al.	NR						NR	12/16
Yong et al.	NR						NR	19/24
Cohorts containing pediatric patients								
Satoshi et al.	4 (5.56%)	10 (13.89%)	33 (45.83%)	21 (29.17%)	2 (2.78%)	2 (2.78%)	NR	17/24
Rutao et al.	5 (4.31%)	15 (12.93%)	48 (41.38%)	34 (29.31%)	10 (8.62%)	4 (3.45%)	NR	20/24
Yuting et al.	36 (30.25%)		83 (69.75%)				7 (10.94%)	18/24

S/C, Surgical/ Conservative; T/S/C, Total/Surgical/Conservative; M/F, Male/Female; PCA, Posterior cerebral artery; SE, Standard error; IQR, interquartile range; NR, Not reported; R, Right; L, Left m, mouth; y, year; The data are presented as mean ± standard deviation, mean (range), and number (percentage).

### 3.2 Baseline information of the studies included in meta-analysis

Following the screening process, a total of seven studies were included in the meta-analysis, involving 393 patients and 649 hemispheres (6, 13, 17–21). All patients are adult AMMD. The study designs included two prospective and five retrospective studies (Table 1). The sample sizes of studies varied between 23 and 103 patients. Except the study by Yuting et al., female patients outnumbered male patients. Most patients had a mean age over 40 years (Table 1). Most studies included both surgical and conservative groups, except for two studies that focused solely on conservative groups. Comorbidities information was available for the vast majority of studies, including diabetes mellitus, hypertension, dyslipidemia, intracranial aneurysm, and smoking, while thyroid disease information was provided in only two studies. Four studies reported information on family history of MMD, and four studies provided information about posterior cerebral artery involvement. All studies described the follow-up duration. Suzuki’s angiographical stage was described in most studies, except for the study by Jeyul et al. A total of five studies were comparative, while two studies were non-comparative. The median scores of non-comparative studies were 13 (12–14), and those of comparative studies were 18 (17–19) (Table 1).

### 3.3 Pooled outcome of AMMD

**Figure 2:**
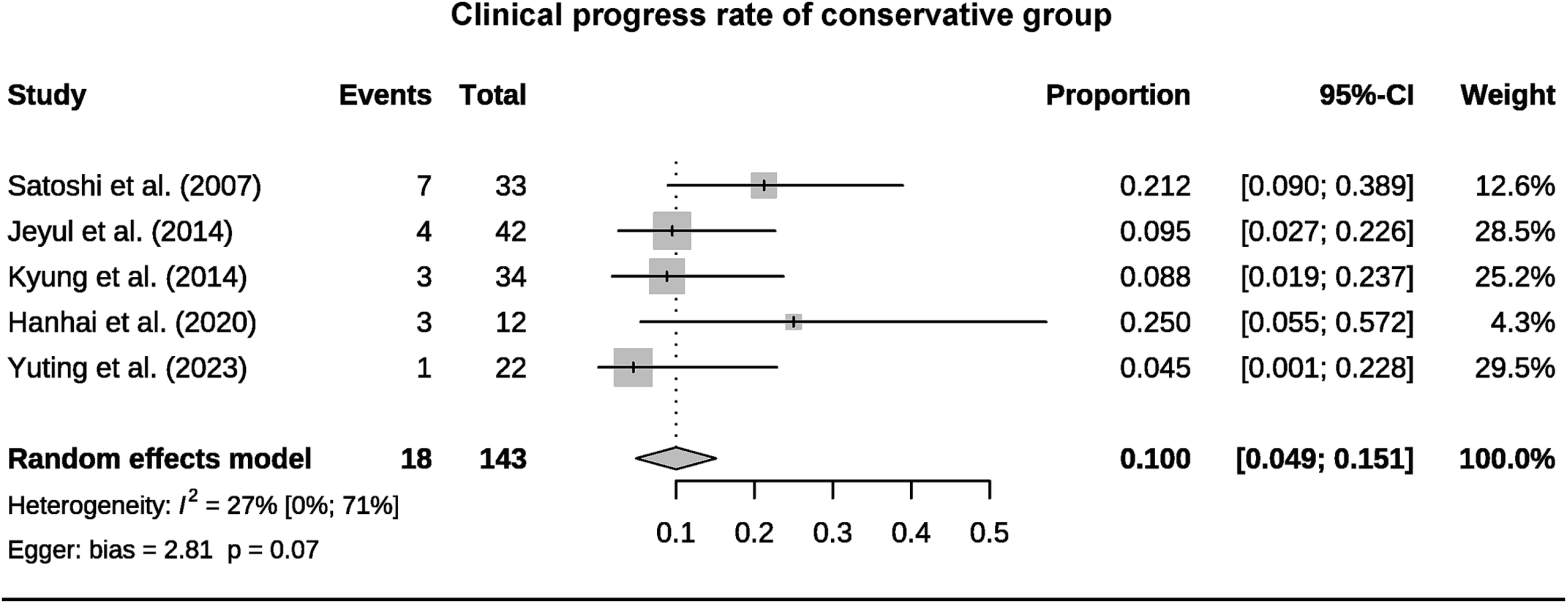
Forest plot for pooled clinical progress rate of the conservative group.

**Figure 3:**
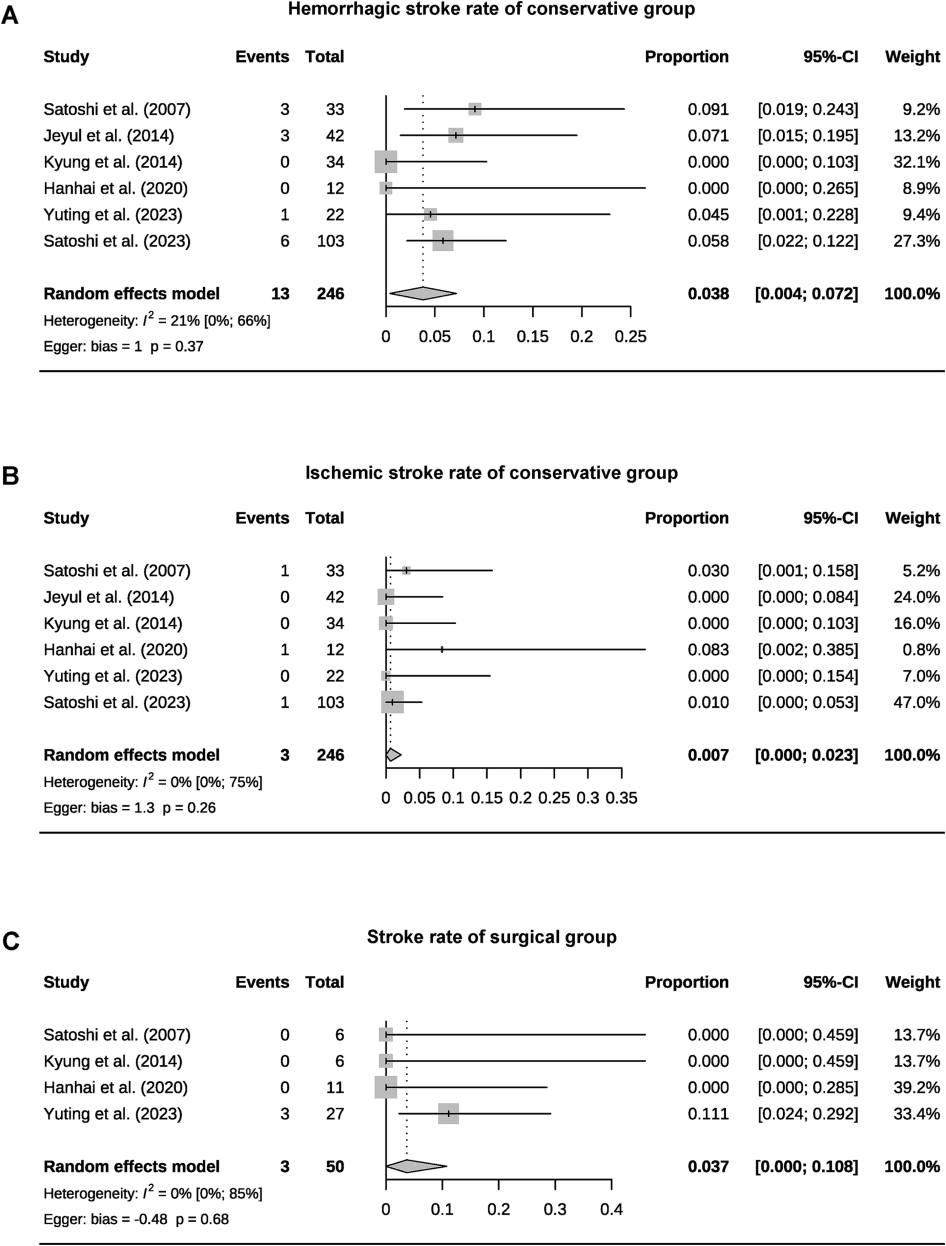
Forest plots for pooled hemorrhagic stroke **(A)**, ischemic stroke rate of the conservative group **(B)**, and stroke rate of surgical group **(C)**.

**Figure 4:**
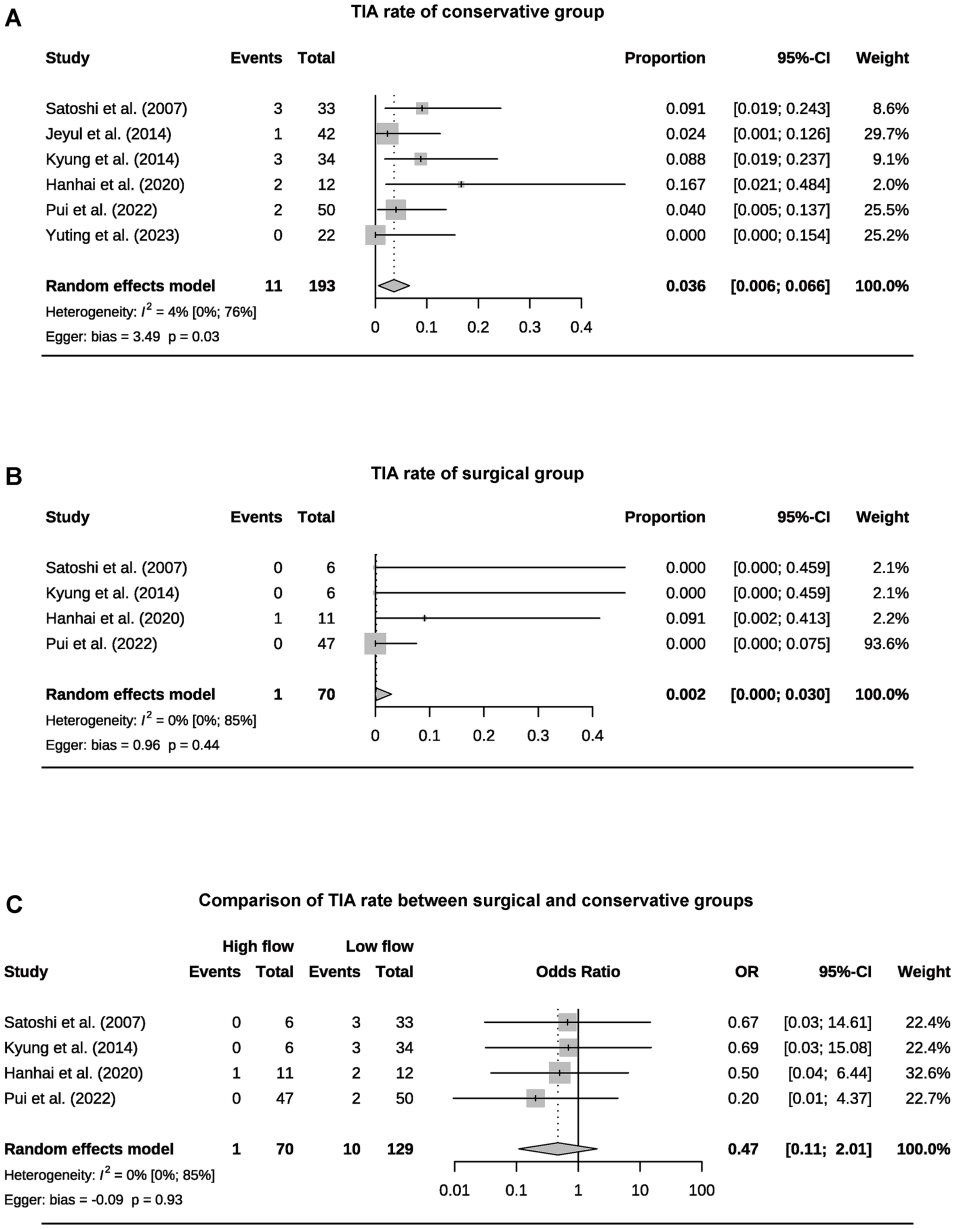
Forest plots for pooled TIA rate of conservative **(A)** and surgical group **(B)**, and comparison of TIA rate between two groups **(C)**.

**Figure 5:**
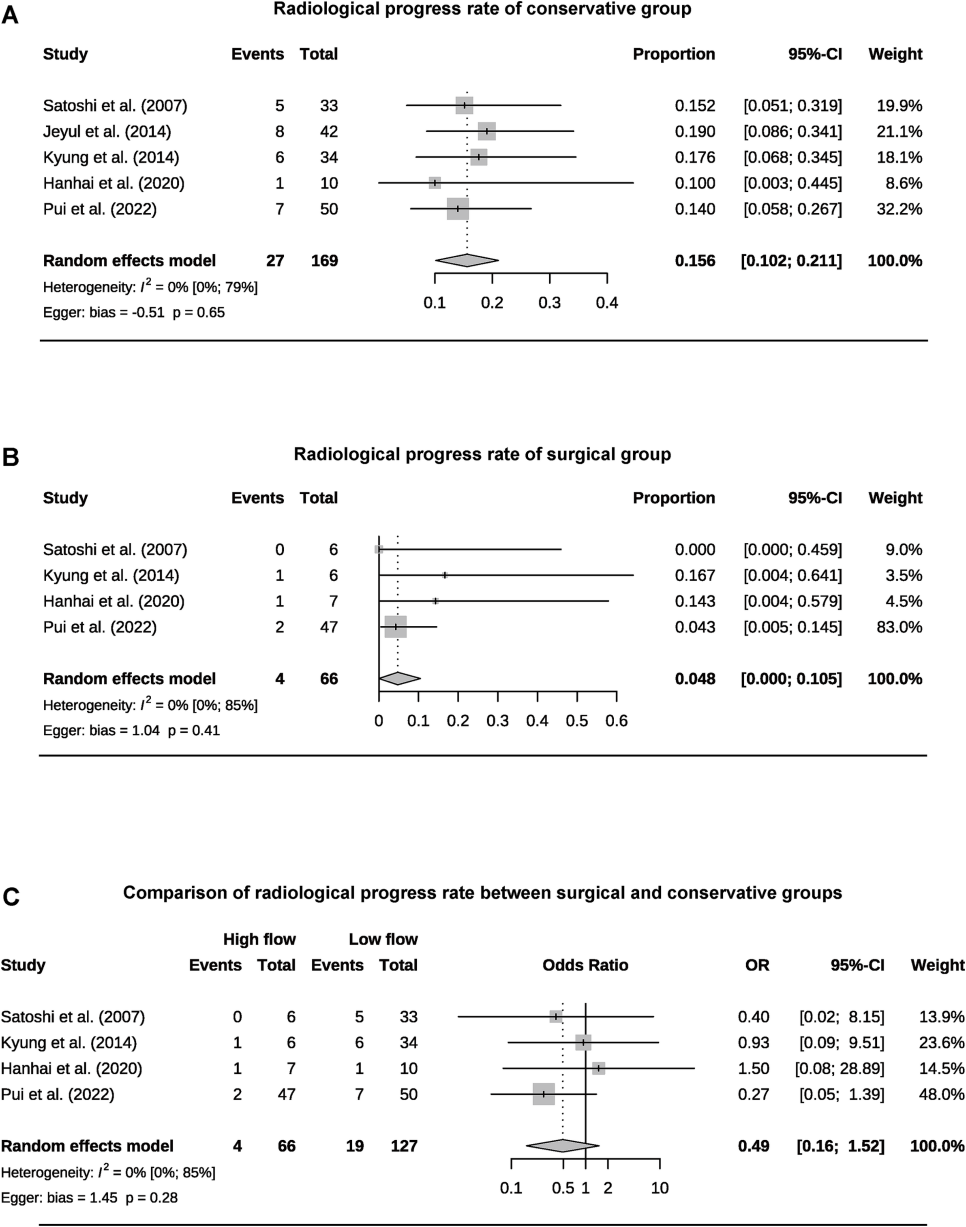
Forest plots for pooled radiological progress rate of conservative **(A)** and surgical group **(B)**, and comparison of radiological progress rate between two groups **(C)**.

A total of 393 patients were included in the analysis, including 97 cases (25%) that underwent surgical treatment and 296 cases (75%) that underwent conservative treatment (Table 3). Among the conservative group, clinical progression was available for five studies, and the pooled rate was 10% (95% CI 4.9–15.1%) (Figure 2). Six studies provided data on stroke in the conservative group, showing the pooled rate of hemorrhagic stroke of 3.8% (95% CI 0.4–7.2%) and ischemic stroke of 0.7% (95% CI 0–2.3%). The pooled stroke incident rate for the surgical group, based on pooled four studies, was 3.7% (95% CI 0–10.8%) (Figure 3). Four studies compared the incident rate of TIA for the surgical vs. conservative groups, and the rate difference was not significant (OR 0.47, 95% CI 0.11–2.01). The pooled TIA rates were 0.2% in the surgical group and 3.6% in the conservative group (Figure 4). The pooled radiological progress rates were 4.8% in the surgical group and 15.6% in the conservative group. The radiological progress comparison between the surgical and conservative groups showed no significant difference (OR 0.49, 95% CI 0.16–1.52), pooled from four studies (Figure 5). The above-pooled results did not show significant heterogeneity. Publication bias was detected in the TIA pooled rate of the conservative group, which might be caused by small sample effects (22).

**Table 3:** Outcome for studies included in meta-analysis.

Study	Surgical					Conservative				
	Clinical progress	TIA	Ischemic stroke	Hemorrhagic stroke	Radiological progress	Clinical progress	TIA	Ischemic stroke	Hemorrhagic stroke	Radiological progress
Satoshi et al.	0	0	0	0	0	7 (21.21%)	3 (9.09%)	1 (3.03%)	3 (9.09%)	5 (15.15%)
Jeyul et al.	/					4 (9.52%)	1 (2.38%)	0	3 (7.14%)	8 (19.05%)
Kyung et al.	0	0	0	0	1 (16.67%)	3 (8.82%)	3 (8.82%)	0	0	6 (17.65%)
Hanhai et al.	1 (9.09%)	1 (9.09%)	0	0	1 (14.29%)	3 (25%)	2 (16.67%)	1 (8.33%)	0	1 (10%)
Pui et al.	NR	0	NR	NR	2 (4.26%)	NR	2 (4%)	NR	NR	7 (14%)
Yuting et al.	NR	NR	3 (11.1%)		NR	1 (4.55%)	0	0	1 (4.55%)	NR
Satoshi et al.	/					NR	NR	1 (0.97%)	6 (5.83%)	NR

NR, Not reported; The data are presented as numbers (percentage).

### 3.4 HSMMD and pediatric cohorts

Three HSMMD studies were conducted at the Seoul National University Hospital, and all patients received conservative treatment. All hemodynamically stable hemispheres included in Young’s study were post-revascularized on the contralateral hemisphere. All three studies were followed up for more than 5 years on average, with an average age of more than 40 years. Moreover, female patients were more than male patients (Table 2). Won et al. reported the highest incidence of stroke, with an ischemic stroke rate of 13.28% and a hemorrhagic stroke rate of 14.11% (Table 4). The other two studies reported a relatively low incidence of stroke during the follow-up. None of the three studies reported a radiological progress rate. Rutao et al. reported 61 patients with AMMD aged 6–59 years (Table 3). Fifty-two received revascularization, while nine received conservative treatment. No stroke occurred during the follow-up of the surgical group, while one hemorrhagic stroke occurred in the conservative group. Satoshi et al.’s study included only one pediatric patient. Yuting et al.’s study included 15 pediatric patients, 40 patients underwent revascularization, and 24 patients underwent conservative treatment. A total of three patients developed stroke during the follow-up of the surgical group. One hemorrhagic stroke occurred in the conservative group.

**Table 4:** Outcomes for overlapping cohorts, HSMMD cohorts, and cohorts containing pediatric patients.

Study	Surgical					Conservative				
	Clinical progress	TIA	Ischemic stroke	Hemorrhagic stroke	Radiological progress	Clinical progress	TIA	Ischemic stroke	Hemorrhagic stroke	Radiological progress
HSMMD in overlapping cohorts										
Won et al.	/					NR	NR	32 (13.28%)	34 (14.11%)	NR
Chang et al.	/					40 (9.11%)	0	10 (2.28%)	30 (6.83%)	NR
Young et al.	/					45 (15.63%)	25 (8.68%)	12 (4.17%)	8 (2.78%)	NR
Overlapping cohorts										
Yudai et al.	/					7 (8.33%)	5 (5.95%)	2 (2.38%)	0	NR
Ryosuke et al.	/					2 (8.33%)	1 (4.17%)	2 (8.33%)	0	5 (20.83%)
Yong et al.	NR	NR	99 (2.94%)	367 (10.9%)	NR	NR	NR	431 (3.41%)	910 (7.21%)	NR
Cohorts containing pediatric patients										
Satoshi et al.	0	0	0	0	0	7 (20.59%)	3 (8.82%)	1 (2.94%)	3 (8.82%)	5 (14.71%)
Rutao et al.	6 (11.54%)	6 (11.54%)	0		NR	3 (33.33%)	2 (22.22%)	0	1 (11.11%)	NR
Yuting et al.	NR	NR	3 (7.5%)		NR	1 (4.17%)	0	0	1 (4.17%)	NR

## 4 Discussion

Due to the limitations imposed by its incidence, research on AMMD is quite limited and primarily concentrated in East Asia, which is associated with the RNF213 coding gene carried by East Asian populations (4). This meta-analysis included seven studies, encompassing a total of 393 patients and 649 hemispheres. The number of female patients exceeded that of male patients, which is consistent with previous research findings (23). The Suzuki stage at the time of diagnosis may have a significant impact on the clinical progress of patients during follow-up (24). This may explain why cohorts with a higher average Suzuki stage tend to have a higher incidence of stroke, as observed in the studies by Yuting et al. (5, 19, 21). Comorbidities can affect the prognosis of patients with MMD. For example, hypertension and diabetes increase the risk of postoperative complications and stroke (25, 26). Moreover, smoking and dyslipidemia are independent risk factors for ischemic stroke in MMD patients (27). Therefore, controlling comorbidities is crucial for patients with MMD, and treatment options for these patients should be more cautious.

Currently, the benefits of antiplatelet therapy (APT) in ischemic MMD remain controversial. However, APT did not increase the risk of hemorrhagic complications. Further high-level evidence is still required to support this conclusion (28, 29). Cilostazol has demonstrated unique advantages compared to other antiplatelet agents (30). The studies by Yuting and Won are the only ones that specifically analyzed the efficacy of APT (5, 21). Unfortunately, APT failed to show a stroke protective effect on either AMMD or HSMMD.

Given the preventive nature of revascularization, the treatment selection for AMMD should be undertaken with greater caution. Currently, evidence-based medical research only supports the benefits of revascularization in symptomatic MMD patients (4, 31). The studies included in the meta-analysis all had an average follow-up period of more than 2 years. The pooled clinical progress rate for the conservative group was 10%, with a hemorrhagic stroke rate of 3.8%, an ischemic stroke rate of 0.7%, and a TIA rate of 3.6%. The stroke rate in the surgical group was 3.7%, while the TIA rate was 0.2%. Revascularization has not demonstrated a significant protective effect on TIA. The clinical progress rate of AMMD without surgical intervention is concerning. Due to the inability to standardize the stroke incidence within a specific follow-up period, this review can only provide a rough estimate of the risk over 2–5 years. The Asymptomatic Moyamoya Registry (AMORE) trial is the only long-term, multicenter, prospective cohort study on the natural course of AMMD. Its interim results indicate an annual stroke risk of 1.4% per person and 0.8% per hemisphere (20). Microbleeds and Grade-2 choroidal anastomosis are predictors of stroke. These results may help optimize treatment choices for AMMD. Yong’s study is the largest retrospective study on AMMD to date. However, due to heterogeneity and differences in the definition of adult age, it was not included in the meta-analysis (32). The study indicates that, compared to conservative treatment, revascularization can reduce mortality in AMMD patients but increase the risk of hemorrhagic stroke and does not provide protection against ischemic stroke. Surgical treatment for AMMD should be approached with caution. Considering that most AMMD patients are middle-aged at the time of diagnosis, their clinical progression rate over their lifetime may be quite significant (33). This suggests that treatment choices based on age can be more specific, with a preference for conservative management in elderly patients (34, 35). Meanwhile, the stroke-protective effect of surgical treatment in younger patients may become more apparent over time. By definition, AMMD will continue to progress even if the patient remains asymptomatic (36). In this review, the pooled radiological progress rate was 15.6% in the conservative group and 4.8% in the surgical group. However, the surgical group did not demonstrate a significant protective effect. This further highlights the importance of developing stroke risk prediction models and establishing regular follow-up guides for AMMD patients.

Currently, only three studies have investigated HSMMD, all of which are from the same institution (5, 37, 38). Won et al. reported an annual stroke incidence of 4.5% per person, with rates of 3.4% in the asymptomatic group, 2.5% for hemorrhagic stroke, and 0.8% for ischemic stroke. Young et al. reported an overall annual stroke risk of 3.0% per person, with ischemic and hemorrhagic stroke rates of 2.5 and 0.5%, respectively. Both studies focused on the natural course of the disease, underscoring the need for future research on the potential effects of APT and revascularization. Pediatric MMD is not within the scope of this systematic review, and a total of three studies, including pediatric AMMD, were included with very limited sample sizes (17, 21, 39). Yuting’s study included 15 pediatric AMMDs, of whom two received conservative treatment, 13 underwent surgical treatment, and all did not experience stroke events (21). More research on pediatric AMMD is needed in the future to guide its treatment options.

This study has several limitations. The majority of the included studies were retrospective and had relatively small sample sizes, particularly in the surgical group. Due to the incidence of AMMD and patients’ concerns regarding preventive surgical treatment, conducting prospective studies remains challenging. Additionally, follow-up durations were not sufficiently long, and standardizing the annual stroke risk was difficult. Studies that mainly focused on HSMMD are all from the Seoul National University College of Medicine, which implies potential numerous overlapping in the cohort, severely limiting the generalizability of results. Given the temporary stability and progressive nature of AMMD and HSMMD, studies with follow-up periods exceeding 10 years may provide more meaningful insights. Furthermore, the majority of studies did not report details of conservative treatment strategies or corresponding clinical outcomes.

In conclusion, AMMD and HSMMD present a concerning risk of clinical and radiological progression over a follow-up period of more than 2 years. However, revascularization has not demonstrated significant benefits within this timeframe. Further high-quality studies are needed to optimize treatment strategies.

## Data Availability

The original contributions presented in the study are included in the article/Supplementary material, further inquiries can be directed to the corresponding author.

## Footnotes

^1^ https://www.crd.york.ac.uk/prospero/display_record.php?RecordID=444432
