# Supplementary material for: Clinical and radiological course of asymptomatic and hemodynamically stable moyamoya disease: a systematic review and meta-analysis
Source: Front Neurol. 2025 Jul 8;16:1626817. doi: 10.3389/fneur.2025.1626817 (PMC12282249; doi:10.3389/fneur.2025.1626817)
Supplement: Supplementary file 2 [file Table_2.docx]

Supplementary Material

# Search queries

| Search number | Query |
| --- | --- |
| 1 | ("Progressive Intracranial Occlusive Arteropathy (Moyamoya)") OR ("Moyamoya Syndrome") OR ("Moya-Moya Disease") OR ("Disease, Moya-Moya") OR ("Moya Moya Disease") OR ("Cerebrovascular Moyamoya Disease") OR ("Moyamoya Disease, Primary") OR ("Disease, Primary Moyamoya") OR ("Moyamoya Diseases, Primary") OR ("Primary Moyamoya Disease") OR ("Primary Moyamoya Diseases") OR ("Moyamoya Disease, Classic") OR ("Classic Moyamoya Disease") OR ("Disease, Classic Moyamoya") OR ("Moyamoya Disease, Secondary") OR ("Secondary Moyamoya Disease") |
| 2 | Moyamoya Disease[MeSH Terms] |
| 3 | #1 OR #2 |
| 6 | ("haemodynamic"[All Fields] OR "hemodynamics"[MeSH Terms] OR "hemodynamics"[All Fields] OR "hemodynamic"[All Fields] OR "haemodynamical"[All Fields] OR "haemodynamically"[All Fields] OR "haemodynamics"[All Fields] OR "hemodynamical"[All Fields] OR "hemodynamically"[All Fields]) AND ("stable"[All Fields] OR "stabled"[All Fields] OR "stables"[All Fields] OR "stabling"[All Fields]) |
| 7 | "asymptomatic"[All Fields] OR "asymptomatically"[All Fields] OR "asymptomatics"[All Fields] |
| 8 | #6 OR #7 |
| 9 | #3 AND #8 |
